# Ferromagnet/Superconductor Hybrid Magnonic Metamaterials

**DOI:** 10.1002/advs.201900435

**Published:** 2019-07-06

**Authors:** Igor A. Golovchanskiy, Nikolay N. Abramov, Vasily S. Stolyarov, Pavel S. Dzhumaev, Olga V. Emelyanova, Alexander A. Golubov, Valery V. Ryazanov, Alexey V. Ustinov

**Affiliations:** ^1^ Moscow Institute of Physics and Technology National Research University 9 Institutskiy per. Dolgoprudny 141700 Moscow Region Russia; ^2^ National University of Science and Technology MISIS 4 Leninsky prosp. 119049 Moscow Russia; ^3^ Institute of Solid State Physics (ISSP RAS) Chernogolovka 142432 Moscow Region Russia; ^4^ Solid State Physics Department Kazan Federal University 420008 Kazan Russia; ^5^ All‐Russian Research Institute of Automatics n.a. N.L. Dukhov (VNIIA) 127055 Moscow Russia; ^6^ National Research Nuclear University MEPhI (Moscow Engineering Physics Institute) 31 Kashirskoye Shosse 115409 Moscow Russia; ^7^ Faculty of Science and Technology and MESA+ Institute for Nanotechnology University of Twente 7500 AE Enschede The Netherlands; ^8^ Faculty of Physics National Research University Higher School of Economics 21/4 Staraya Basmannaya Str. 105066 Moscow Russia; ^9^ Physikalisches Institut Karlsruhe Institute of Technology 76131 Karlsruhe Germany

**Keywords:** ferromagnetic resonance, magnonic crystals, spin waves, superconductivity

## Abstract

In this work, a class of metamaterials is proposed on the basis of ferromagnet/superconductor hybridization for applications in magnonics. These metamaterials comprise of a ferromagnetic magnon medium that is coupled inductively to a superconducting periodic microstructure. Spectroscopy of magnetization dynamics in such hybrid evidences formation of areas in the medium with alternating dispersions for spin wave propagation, which is the basic requirement for the development of metamaterials known as magnonic crystals. The spectrum allows for derivation of the impact of the superconducting structure on the dispersion: it takes place due to a diamagnetic response of superconductors on the external and stray magnetic fields. In addition, the spectrum displays a dependence on the superconducting critical state of the structure: the Meissner and the mixed states of a type II superconductor are distinguished. This dependence hints toward nonlinear response of hybrid metamaterials on the magnetic field. Investigation of the spin wave dispersion in hybrid metamaterials shows formation of allowed and forbidden bands for spin wave propagation. The band structures are governed by the geometry of spin wave propagation: in the backward volume geometry the band structure is conventional, while in the surface geometry the band structure is nonreciprocal and is formed by indirect band gaps.

## 1 Introduction

Magnonics is a rapidly growing field of research that studies materials, structures, devices, and circuits for transfer and processing of microwave signals via spin waves.^1^, ^2^, ^3^, ^4^, ^5^, ^6^, ^7^, ^8^, ^9^ In magnonics, one of the main building blocks are the so‐called magnonic crystals (MCs).^9^, ^10^, ^11^, ^12^, ^13^, ^14^ MCs are magnetic metamaterials with periodic modulation of any magnetic parameter that is relevant to the dispersion of spin waves: external magnetic field, saturation magnetization, exchange properties, magnetic anisotropy, film thickness, mechanical stress, etc. MCs can be understood as the magnetic counterpart of photonic crystals, their major characteristic is the presence of allowed and forbidden bands for spin wave propagation. MCs are currently considered for application as waveguides,^2^, ^15^ filters,^16^ grating couplers,^17^ and in data processing devices.^12^ The main advantages of MCs for applications are the tunability of the band structure by external magnetic field, convenient micro and sub‐microscales, and the microwave frequency range of operation.

Currently, a rich variety of approaches exists for development of 1D and 2D MCs in planar geometry. This variety includes straightforward ways, such as periodic grooving or thinning of ferromagnetic films,^18^, ^19^, ^20^ as well as more sophisticated techniques. For instance, employment of current‐carrying microstructures,^21^ development of bi‐component MCs,^13^, ^22^, ^23^, ^24^ dot^25^ and antidot^9^, ^15^, ^26^ ferromagnetic lattices, nanowire lattices,^27^ engineering of systems with antiferromagnetic coupling,^28^ and systems with periodic Dzyaloshinskii–Moriya interactions^29^ have been reported. Various types of MCs are developed pursuing miniaturization of magnonic devices and tunability of their operation bands.

In this work, we propose to compose MCs by hybridizing ferromagnetic (F) films with periodic superconducting (S) structures, i.e., as S/F hybrids. Currently, hybridization of ferromagnets with superconductors for magnonics is gaining a momentum. In particular, in superconducting‐proximity‐coupled Nb/Ni_80_Fe_20_/Nb three‐layers in in‐plane fields, a substantial reduction of the ferromagnetic resonance (FMR) field by μ_0_ *H* ≈ 30–80 mT (and corresponding enhancement of the FMR frequency by several GHz) is reported.^30^, ^31^ This reduction of the resonance field by superconducting phenomenon is believed to be attributed to the generation of unconventional spin‐triplet superconductivity^30^ or to an interplay of the ferromagnetic layer with superconductor‐induced magnetic flux.^31^ Also, a number of effects were reported for proximity‐decoupled superconductor/ferromagnet bi‐layer systems in out‐of‐plane magnetic fields. On elementary level in such systems, an interplay of magnetization dynamics in a ferromagnet with the critical magnetic state of a superconductor takes place,^32^ which results in a complex hysteresis behavior of the FMR absorption. On the other side, coupling of a ferromagnetic film with the ideal hexagonally ordered superconducting vortex lattice produces periodic perturbations of magnetic order in the film and facilitates formation of forbidden bands for spin wave propagation.^33^ In such magnonic architecture, forbidden bands are opened at Brillouin wavenumbers that correspond to the period of the flux‐line lattice, and therefore, can be adjusted by changing the out‐of‐plane component of magnetic field. Moreover, moving superconducting vortices can also be employed for radiation of magnons in superconductor‐ferromagnet multilayers.^34^ In case of proximity‐decoupled superconductor/ferromagnet bi‐layers in in‐plane fields, it was demonstrated that coupling of spin waves with a superconductor results in enhanced phase velocity of spin waves.^35^, ^36^ The enhancement occurs due to screening of AC magnetostatic stray fields by a superconductor, and can be viewed as the interaction of spin waves with an ideal conductor,^37^, ^38^, ^39^, ^40^ or with the Meissner screening currents.^35^, ^36^

We propose to employ capabilities of superconductors to modify dynamic properties of ferromagnets for development of a medium with periodically modulated spin wave dispersion. As discussed in this work, dispersion of such metamaterials exhibits magnonic band structures. Dynamic behavior of MCs is determined by the geometry of spin wave propagation. In addition, a nonlinear response of hybrid metamaterials on magnetic field is noted. Development of MCs by hybridizing ferromagnetic films with superconductors may appear to be effective for application in cryogenic temperatures (see references in ref. [32]), it paves the way for design of tunable MCs on microscales.

## 2 Results and Discussions

### 2.1 Experimental System

**Figure 1:**
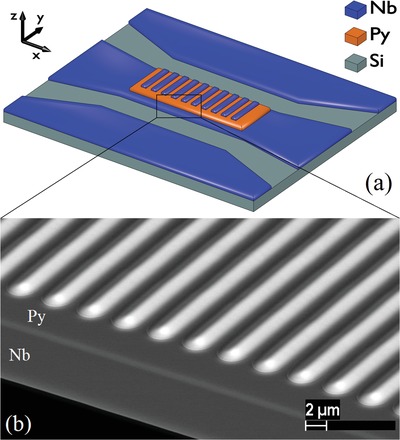
a) Schematic illustration of the investigated test chip (not to scale). A 50 nm thick Py film (shown in orange) is placed on top of the central conducting line of Nb CPW (shown in blue). Nb stripes of thickness 0.7 µm and width 3 µm are placed regularly on top of Py thin film with the period 4 µm. Magnetic field *H* is applied along *x*‐direction and excitation microwave field is applied along *y*‐direction. b) Scanning electron microscopy image of the fabricated structure taken with back‐scattered electrons and a 60°‐tilt of the sample table.

The investigated hybrid system is illustrated in **Figure** **1**. The system consists of a regular superconducting niobium (Nb) structure placed on top of ferromagnetic Ni_80_Fe_20_ permalloy (Py) thin film. The superconducting periodic structure is represented by an array of Nb stripes of dimensions *X* × *Y* × *Z* = 3 × 130 × 0.7 µm^3^ placed with the period *a* = 4 µm along the *x*‐axis. The stripes have a triangular rather than a rectangular cross‐section in *x*–*z* plane with the base 3 µm and the height 0.7 µm (see Figure 1b and the Supporting Information). The triangular cross‐section is formed due to the off‐axis deposition of magnetron sputtered Nb film on the substrate, which is primed for the lift‐off process, i.e., that is covered with a patterned photo‐resist. The array of stripes is placed on top of 1100 × 130 µm^2^ Py rectangle film of thickness *d* = 50 nm. The Py/Nb sample is placed on top of 150 µm wide central stripe of the 50 Ω impedance superconducting Nb coplanar waveguide (CPW) formed on Si substrate. A 5 nm thick AlO_*x*_ insulating layer is deposited between superconducting and ferromagnetic layers in order to avoid the superconducting proximity effect. The measured superconducting critical temperature of Nb CPW is *T*_c_ ≈ 9 K. The superconducting critical temperature of Nb stripes is expected to be reduced due to contamination from organic resist during deposition and is expected to be *T*_c_ > 8 K. Magnetic field *H* is applied along the *x*‐direction, i.e., the so‐called backward volume (BV) geometry^18^, ^41^ is realized.

The FMR absorption spectroscopy was performed using the vector network analyzer (VNA; the so‐called VNA‐FMR approach).^42^, ^43^, ^44^, ^45^ In this work, the same experimental setup was used for investigation of the resonant absorption and the same sample layout as in refs. [35,45].

### 2.2 FMR Spectrum of Hybrid Metamaterials

**Figure 2:**
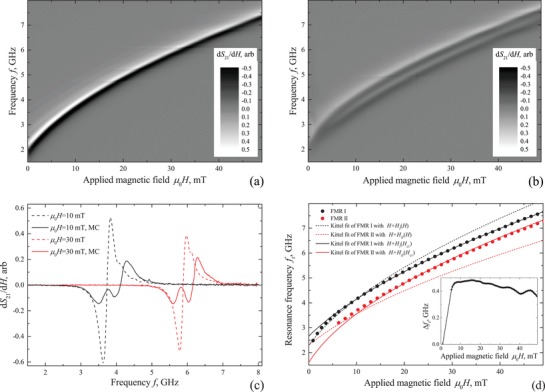
a,b) Transmission spectra *dS*_21_(*f*, *H*)/*dH* of the a) pristine Py film and b) MC sample measured at 4 K. c) Cross‐sections *dS*_21_( *f*  )/*dH* of spectra at few selected magnetic fields *H*. d) Dependencies of FMR frequency on magnetic field *f*_r_(*H*). Experimental *f*_r_(*H*) curves extracted from (b) are shown with dots. Solid and dashed lines show the Kittel fit with the screening effect incorporated. The inset in (d) shows the dependence of the frequency difference between resonance curves on magnetic field Δ*f*_r_(*H*).

**Figure** **2**a,b shows transmission spectra *dS*_21_(*f*, *H*)/*dH* of the studied sample at *T* = 4 K. For better appearance of experimental data, both measured spectra *S*_21_(*f*, *H*) have been first normalized with *S*_21_( *f*  ) at µ_0_ *H* = 0.5 T, and then differentiated numerically in respect to *H*. Spectra were measured for two samples: for a pristine ferromagnetic film prior to deposition of Nb stripes (Figure 2a), and for the same Py film with deposited Nb stripes (Figure 2b), referred to as the MC sample. Field‐dependent spectral lines in Figure 2a,b correspond to FMR curves *f*_r_(*H*). Figure 2c compares cross‐sections of spectra *dS*_21_(*f*)/*dH* obtained before and after deposition of Nb stripes.

FMR curve *f*_r_(*H*) of the pristine sample follows the typical Kittel dependence for thin ferromagnetic films in in‐plane magnetic fields^41^

(1) $${\left(2\pi f_{r}/\mu_{0}\gamma \right)}^{2}=\left(H+H_{a}\right)\left(H+H_{a}+M_{\mathrm{eff}}\right)$$

where µ_0_ is the vacuum permeability, γ = 1.856 × 10^11^ Hz T^−1^ is the gyromagnetic ratio for Py, *H*_a_ is the anisotropy field, and *M*_eff_ is the effective saturation magnetization that includes the actual saturation magnetization *M*_s_ and the out‐of‐plane magnetic anisotropy. The fit of the FMR curve in Figure 2a with Equation (1) yields the following parameters typical for Py: µ_0_ *H*_*a*_ = 4 mT and µ_0_ *M*_eff_ = 1.14 T. Note that conventionally the value of the effective magnetization for Py is about 1 T. Slightly higher value of *M*_eff_ in this work can be explained by enhancement of the saturation magnetization in low temperatures in accordance with the Bloch theory (*M*_s_∝ − *T* ^3/2^), as well as by possible presence of growth‐induced or thermal‐expansion‐mediated anisotropies.^32^

The spectrum of the MC sample (Figure 2b,c) is qualitatively different when measured below the superconducting critical temperature of Nb. Application of superconducting Nb stripes results in splitting of the FMR signal into two spectral lines. Experimental resonance curves *f*_r_(*H*) for the MC sample are given in Figure 2d with dots. As follows from Figure 2b,d, the distance between resonance curves Δ*f*_r_ increases progressively from 0 up to ≈0.45 GHz when magnetic field is changed from 0 to ≈10 mT, and then decreases slowly from Δ*f*_r_ ≈ 0.45 GHz to Δ*f*_r_ ≈ 0.4 GHz when magnetic field is further increased from µ_0_ *H* ≈ 10 mT to 50 mT (see the inset in Figure 2d). Importantly, at *T* > *T*_c_ the FMR spectrum of the MC sample consists of a single resonance line and in general reproduces one of the pristine sample (see the Supporting Information). Therefore, the splitting of the FMR signal is clearly associated with superconductivity of Nb stripes.

We state that the very basic property of superconductors, i.e., the expulsion of the magnetic field from a superconductor, is responsible for the splitting. Indeed, when superconducting stripes screen‐out the magnetic field from their interior it increases *H* outside of stripes in their vicinity due to the demagnetizing effect.^46^ Therefore, we conclude that the higher‐frequency stronger FMR line in Figure 2b,d is a result of the FMR absorption by the area of Py film located directly under the Nb stripes where the field is enhanced (referred to as area I), while the lower‐frequency weaker FMR line is a result of the FMR absorption by the area of Py film located between Nb stripes (referred to as area II). In general, a magnetic structure that consists of two periodic areas with alternating FMR conditions is referred to as the MC, for such structure a discontinuity of the spin wave spectrum is expected at the Brillouin wavenumbers of the structure ∝1/*a*. Below we analyze in details the impact of superconducting stripes on the FMR spectrum of the MC sample.

The curve Δ*f*_r_(*H*) in Figure 2d reflects the dependence of the superconducting critical state of Nb stripes on the magnetic field. At low magnetic fields µ_0_ *H* < 10 mT, where Δ*f*_r_(*H*) grows rapidly with *H*, Nb stripes are at the ideal diamagnetic (i.e., Meissner) state. At the Meissner state, the magnetic flux is expelled from a superconductor by the circulating Meissner screening currents, and, therefore, the effect of superconducting demagnetizing field on FMR is simply proportional to the applied magnetic field. Upon increasing the magnetic field the Meissner state is terminated at µ_0_ *H*_c1_ ≈ 10 mT, known as the first superconducting critical field. This estimate includes the demagnetizing factor of Nb stripes. At *H* > *H*_c1_, magnetic flux starts to penetrate in the form of Abrikosov vortices, the ideal diamagnetic response of Nb stripes on the external magnetic field is ceased. However, the Meissner screening currents persist since both FMR lines remain separated. Such behavior implies a nonlinear dependence of the spin wave dispersion on magnetic field. Note that typically in Nb the first critical field µ_0_ *H*_c1_ ∼ 10–100 mT. In this work, low µ_0_ *H*_c1_ ≈ 10 mT indicates a nonexcellent quality of Nb that is a result of deposition of Nb onto the substrate that is covered with organic resist.

Importantly, the spectrum of the MC sample in Figure 2b is almost reversible in respect to the *H*‐axis. Reversibility of the FMR spectrum indicates reversibility of magnetization of superconducting Nb stripes. Reversibility of magnetization of a type‐II superconductor points toward inapplicability of the Bean critical state model,^47^, ^48^ which correlates the magnetization of hard type‐II superconductors with the pinning of Abrikosov vortices. In our case, reversibility of magnetization of Nb stripes is promoted by the vortex shaking mechanism^49^, ^50^, ^51^, ^52^, ^53^, ^54^ when alternating magnetic fields depin vortices and prevent formation of the Bean gradient of magnetic flux. Note, that the reversible behavior of the FMR spectrum is in contrasts with one for S/F film structures in out‐of‐plane magnetic fields.^32^

**Figure 3:**
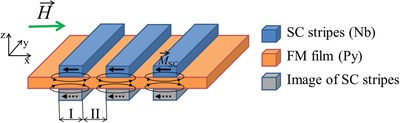
Estimation of additional DC magnetic field that is induced by the Meissner‐screened Nb stripes. Each superconducting stripe possesses the magnetic moment ${\vec{M}}_{\mathrm{sc}}(H)=-\vec{H}$ indicated with black arrows. Each magnetized superconducting stripe is accompanied by its mirrored image in respect to the surface of the waveguide that also possesses the same magnetic moment ${\vec{M}}_{\mathrm{sc}}(H)=-\vec{H}$ indicated with black dashed arrows. Magnetostatic stray fields of these moments (indicated with black lines) modulate periodically the DC magnetic field in Py film along the *x*‐axis.

The impact of the superconducting structure on the FMR spectrum can be quantified by the estimation of the magnetostatic stray fields in the Py film induced by the superconducting structure, as illustrated in **Figure** **3**. Superconducting stripes, being treated as ideal diamagnets, possess magnetization ${\vec{M}}_{\mathrm{sc}}(H)=-\vec{H}$ everywhere inside stripes. In addition, since the Py/Nb sample is placed on top of the central line of superconducting Nb CPW (see Figure 1), each magnetized superconducting stripe is accompanied by its mirrored image in respect to the surface of the waveguide.^35^, ^45^ Magnetostatic stray fields of these magnetizations modulate periodically the DC magnetic field in the Py film along the *x*‐axis. We estimate that the actual average magnetic field in Py film along the *x*‐axis that is induced by superconducting stripes in area I is *H*_I_(*H*) ≈ +0.18*H*, and in area II is *H*_II_(*H*) ≈ −0.27*H*. Magnetostatic field produced by Nb stripes in Py film along the *z*‐axis is compensated‐out by the *z*‐component of image fields. Dashed curves in Figure 2d show the Kittel FMR lines calculated using Equation (1) with µ_0_ *H*_a_ = 5 mT, the same *M*_eff_ as for the pristine sample, and with *H* substituted by *H* + *H*_I_ and *H* + *H*_II_. Both curves diverge upon increasing the magnetic field and fit well to corresponding FMR lines at low magnetic fields µ_0_ *H* ≲ 10 mT, which indicates applicability of the employed estimation of magnetization ${\vec{M}}_{\mathrm{sc}}=-\vec{H}$ for superconductors at the Meissner state. At µ_0_ *H* > 10 mT the fit can be obtained by fixing the diamagnetic response of superconductors at *H* = *H*_c1_. Solid curves in Figure 2d show the Kittel FMR lines calculated using Equation (1) with the same magnetic parameters as for the dashed lines, and with *H* substituted by *H* + *H*_I_(*H*_c1_) and *H* + *H*_II_(*H*_c1_). Both curves fit well to corresponding FMR lines at high magnetic fields µ_0_ *H* > 10 mT, which indicates applicability of the employed estimation of reversible magnetization ${\vec{M}}_{\mathrm{sc}}=-{\vec{H}}_{\mathrm{c1}}$ for superconductors at the penetrated state at *H* > *H*_c1_.

### 2.3 Magnonic Band Structures of Hybrid Metamaterials

**Figure 4:**
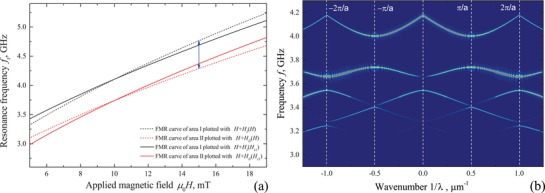
Characteristics of magnetization dynamics for the hybrid MC in BV geometry. The MC consists of 100 nm thick Py film and an array of rectangular superconducting stripes with cross‐section *X* × *Z* = 0.5 × 0.3 µm^2^ located with the period *a* = 1 µm along the *x*‐axis. a) Dependencies of FMR frequency on magnetic field *f*_r_(*H*) in areas I and II of the MC calculated using Kittel formula. The blue arrow indicates the difference of approximately 0.5 GHz between FMR frequencies of area I and II, respectively, at µ_0_ *H* = 15 mT. b) The color‐coded band structure of the S/F hybrid MC at µ_0_ *H* = 15 mT. The maximum of the Fourier transform is coded with red.

The specified estimation for magnetization of superconductors can be employed in micromagnetic simulations of the magnonic band structure. However, investigation of the band structure of the fabricated sample (Figure 1) is unpromising and computationally heavy mainly due to large period *a* of the structure. Therefore, a structure with different dimensions is considered for numerical simulations. We consider the hybrid MC that is represented by a 100 nm thick Py ferromagnetic film with lateral dimensions *X* × *Y* = 900 × 900 µm^2^ and an array of superconducting stripes of dimensions *X* × *Y* × *Z* = 0.5 × 900 × 0.3 µm^3^ placed with the period *a* = 1 µm along the *x*‐axis. In this geometry, the averaged magnetic field in Py film along the *x*‐axis that is induced by superconducting stripes in area I is *H*_I_(*H*) ≈ +0.14*H*, and in area II is *H*_II_(*H*) ≈ −0.14*H*. Dependencies of resonance frequency on magnetic field for this MC are given in **Figure** **4**a.

Figure 4b shows the simulated band structure of the S/F hybrid MC in the BV geometry, i.e., in the geometry of the experiment (Figure 1), at µ_0_ *H* = 15 mT. Details of simulations are given in Section 4. Calculating the spectrum in Figure 4, only the DC screening of applied magnetic field by superconducting stripes is considered. Also, screening by the underlying superconducting surface is not considered. The band structure in Figure 4b is typical for an MC in BV geometry. The forbidden bands are opened at Brillouin wavenumbers ∝1/*a* that are indicated with dashed lines. The first two gaps are opened at frequencies *f* = 3.87 and 3.6 GHz with the width of band gaps Δ*f* = 0.26 and 0.1 GHz, respectively. Band gaps with larger numbers, i.e., at lower frequencies, show smaller gap width Δ*f*. In this geometry, the nonlinear response of superconducting stripes on magnetic field is expected to manifest itself in widening of the forbidden bands with increasing *H* at *H* < *H*_c1_ and narrowing of the bands with increasing *H* at *H* > *H*_c1_, in accordance with Figures 2d and 4a.

In the magnetostatic surface spin wave (MSSW) geometry,^18^, ^41^ if superconducting stripes are aligned along the *x*‐direction in Figures 1 and 3, the interaction of magnetization dynamics with superconducting stripes is different. In this geometry, the DC magnetic field in Py film that is induced by superconducting stripes along the direction of external field is negligible due to small demagnetizing factor of stripes. This leads to the absence of any effect of Nb stripes on the FMR spectrum (see the Supporting Information).

**Figure 5:**
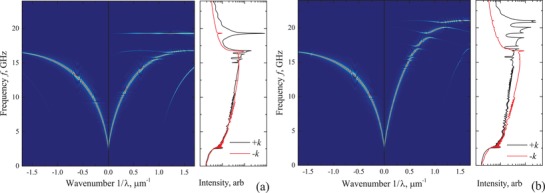
Color‐coded band structures of hybrid MCs in MSSW geometry. MCs consist of 100 nm thick Py film and an array of rectangular superconducting stripes with cross‐sections a) *Y* × *Z* = 0.3 × 0.3 µm^2^ and b) *Y* × *Z* = 0.9 × 0.3 µm^2^ located with the period *a* = 1 µm along the *y*‐axis. The maximum of the Fourier transform is coded with red. Right panels show the dependence of the integrated Fourier transform along the wavenumber axis on frequency for positive (+*k*) and negative (−*k*) directions of spin wave propagation.

However, in the MSSW geometry superconducting stripes can interplay with spatially nonuniform precession of magnetic moments, as in case of spin waves. The result of such interaction is given in **Figure** **5**, where simulated band structures of two S/F hybrid MCs in MSSW geometry are shown. The S/F hybrid structures in Figure 5 are represented by the same 100 nm thick Py ferromagnetic film and arrays of superconducting stripes of dimensions *X* × *Y* × *Z* = 900 × 0.3 × 0.4 µm^3^ (Figure 5a) and *X* × *Y* × *Z* = 900 × 0.9 × 0.4 µm^3^ (Figure 5b) located with the same period *a* = 1 µm along the *y*‐axis. Right panels in Figure 5 show the dependence of the integrated amplitude of the spectrum along the wavenumber axis on frequency for positive (+*k*) and negative (−*k*) directions of spin wave propagation. This amplitude correlates with the spin wave transmission characteristics.

Spectra of S/F hybrids in MSSW geometry in Figure 5 are more sophisticated than one in BV geometry and possess several features. First, a clear nonreciprocity is observed, spectral lines with positive wavenumbers reveal the forbidden bands while spectral lines with negative wavenumbers are continuous. Nonreciprocity of the band structure is a consequence of a general nonreciprocity of the MSSW mode:^36^, ^41^, ^55^, ^56^ the wave energy is localized at a particular surface of the film depending on the direction of wave propagation in respect to the applied magnetic field. Localization of superconducting structure on one side of ferromagnetic film results in effective interaction of magnetization dynamics with superconducting stripes for positively propagating spin waves exclusively. The nonreciprocal spin wave transmission characteristics suggest application of such MCs as one‐way waveguides and filters. In addition, the band gaps in Figure 5 are opened away from the Brillouin wavenumbers ∝1/*a*. Such band gaps are referred to as the indirect band gaps and appear as a consequence of nonreciprocity of the dispersion law.^19^, ^23^, ^57^, ^58^ A quick visual comparison of two S/F hybrids with the same lattice period but different sizes of superconducting stripes implies that position of band gaps in (1/λ, *f*) coordinates and the width of band gaps depend on dimensions. Unlike in case of the BV geometry (Figure 4), band gaps with larger numbers that are located at higher frequencies show larger width Δ*f*; the maximum Δ*f* = 2.5 GHz is observed at *f* = 18.0 GHz in Figure 5a, and the maximum Δ*f* = 0.7 GHz is observed at *f* = 20.4 GHz in Figure 5b. This effect is a result of a more efficient screening for shorter spin waves, which produces larger difference in dispersion properties between areas I and II. In the MSSW geometry, nonlinear dependence of magnetization of superconductor on magnetic field is expected to manifest itself in dependence of the band structure on the amplitude of excitation field.

## 3 Conclusion

Summarizing, in this work we have considered magnetization dynamics in ferromagnet/superconductor hybrid MCs, which consist of a ferromagnetic film coupled inductively to a superconducting periodic microstructure. Studying the FMR spectrum of the hybrid, we have defined the actual contribution of the superconducting periodic subsystem to magnetization dynamics, that is the diamagnetic response of the superconductor. In addition, we have observed the correlation of the FMR spectrum with the superconducting critical states, have identified the Meissner state and the vortex‐penetrated state. Reversibility of the FMR spectrum suggests an action of the vortex shaking mechanism on superconducting vortices.

Dispersions of spin waves in hybrid MCs have been considered in in‐plane geometries. In the BV geometry, a conventional band structure is formed with band gaps that are opened at the Brillouin wavenumbers. The band structure is formed mainly due to screening of the external DC magnetic field by superconducting stripes and due to formation of corresponding spatial variation of the DC field. In the MSSW geometry, the dispersion is nonreciprocal and the band structure is formed by indirect band gaps. This band structure is formed mainly due to screening of the AC component of stray fields of precessing magnetic moments. In general, the proposed metamaterials offer a simple tunability of their dispersions by adjusting geometrical parameters of the superconducting periodic structure, or the orientation of the spin wave propagation. The dependence of screening capabilities of superconducting elements on the magnetic field points toward a nonlinear spin wave dynamics in S/F magnonic metamaterials.

As a final remark, we note limitations for application of superconductors for magnonic metamaterials. Operation of superconductors as diamagnets is possible at temperatures below the superconducting critical temperature and magnetic fields below the upper superconducting critical field. The operation frequency should remain below the superconducting gap frequency. Dimensions of superconductors should remain above the London penetration depth λ_L_. Small typical scales of λ_L_ pave the way for the development of MCs with micro and sub‐microscaled periodicity.

## 4 Experimental Section

The superconducting waveguide was fabricated on Si substrate out of magnetron sputtered 100 nm thick Nb film with superconducting critical temperature *T*_c_ ≃ 9 K using optical lithography and plasma‐chemical etching in CF_4_ + O_2_ plasma. The base pressure in the growth chamber prior deposition was 5 × 10^−9^ mBar. Prior to deposition of Nb, the substrate was plasma‐cleaned at *P*_Ar_ = 2 × 10^−2^ mBar, 60 W RF power, and 500 V DC voltage. During deposition of Nb, the argon pressure, RF power, deposition rate, and DC voltage were 4 × 10^−3^ mBar, 200 W, 2.2 Å s^−1^, and 200 V, respectively. Py thin film was deposited using successive magnetron RF‐sputtering of Py alloy target and the double resist lift‐off technique. During deposition of Py, the argon pressure, RF power, deposition rate, and DC voltage were 4 × 10^−3^ mBar, 200 W, 1.5 Å s^−1^, and 450 V, respectively. Periodic Nb superconducting stripes were deposited using successive magnetron RF‐sputtering and the double resist lift‐off technique. AlO_*x*_ insulating layer was deposited sputtering Al elemental target in Ar + O_2_ atmosphere with 115 sccm of Ar flow and 35 sccm of O_2_ flow. During AlO_*x*_ deposition, Ar + O_2_ pressure, RF power, deposition rate, and DC voltage were 4 × 10^−3^ mBar, 200 W, 0.6 Å s^−1^, and 510 V, respectively.

Numerical analysis was performed using micromagnetic simulations^59^, ^60^ following refs. [24,61–63]: a magnetic field pulse of a sinc temporal and spatial profiles was applied locally to a simulated ferromagnet orthogonally to the DC magnetic field, and the evolution of local magnetic moments in a ferromagnet $\vec{M}(\vec{r},t)$ was recorded. The maximum of the amplitude of the space‐time Fourier transform of $\vec{M}(\vec{r},t)$ provided the dispersion $f(\vec{k})$. In order to avoid reflections, an exponential Gilbert damping profile was set near the boundaries of the film. The following micromagnetic parameters were used for calculations (Figures 4 and 5), that were typical for Py:^35^, ^45^ µ_0_ *H* = 15 mT (Figure 4) and µ_0_ *H* = 3.8 mT (Figure 5), µ_0_ *M*_s_ = 1.14 T, µ_0_ *H*_a_ = 3.1 mT, γ = 1.856 × 10^11^ Hz T^−1^, the exchange stiffness constant *A* = 1.3 × 10^−11^ J m^−1^. Dimensions of the simulated Py film were *L* × *W* × *d* = 900 × 900 × 0.1 µm^3^. The excitation pulse was of a sinc temporal profile with the maximum frequency *f*_max_ = 8 GHz (Figure 4) and *f*_max_ = 30 GHz (Figure 5), of a sinc spatial profile with the the maximum wave‐vector *k*_max_ = 2π/800 nm^−1^ and of an amplitude of 0.001*M*_s_. It was sufficient to perform the micromagnetic simulation employing a 1D mesh in order to capture correctly the magnetostatic spin wave dispersion.

For the calculation of Figure 4, the effect of the superconducting structure was accounted by the calculation of distribution of the DC magnetostatic stray field induced by the diamagnetic magnetization of superconductors in Py film. Both *x*‐ and *z*‐components of the magnetostatic field were considered. The magnetostatic problem of S/F hybrids in MSSW geometry (Figure 5) was different from one in BV geometry since the effect from DC screening of the external magnetic field is negligible. This problem was treated as magnetostatic interaction of a ferromagnet with an ideal diamagnet with oscillating magnetization ${\vec{M}}_{\mathrm{sc}}=-\vec{H}$ that is induced by alternating stray fields. Numerical implementation of the magnetostatic problem of S/F hybrids in micromagnetic simulations was executed by the incorporation of an intermediate step for the calculation of magnetization ${\vec{M}}_{\mathrm{sc}}$, which was a result of the diamagnetic response on both the DC external field and AC stray fields of a ferromagnet, and then by the calculation of the total dipole–dipole component of the effective field at each time step of integration of the Landau–Lifshitz–Gilbert equation. Note that this numerical approach is different from the method of images^35^, ^36^ employed earlier for study of magnetization dynamics in a ferromagnet placed on top of the infinite superconducting surface.

## Conflict of Interest

The authors declare no conflict of interest.

## Supporting information

Supplementary

## References

[1] A. V. Chumak , V. I. Vasyuchka , A. A. Serga , B. Hillebrands , Nat. Phys. 2015, 11, 453.

[2] B. Lenk , H. Ulrichs , F. Garbs , M. Munzenberg , Phys. Rep. 2011, 507, 107.

[3] G. Csaba , A. Papp , W. Porod , Phys. Lett. A 2017, 381, 1471.

[4] A. Haldar , C. Tian , A. O. Adeyeye , Sci. Adv. 2017, 3, e1700638. 28776033 10.1126/sciadv.1700638 PMC5521997

[5] Y. Kajiwara , K. Harii , S. Takahashi , J. Ohe , K. Uchida , M. Mizuguchi , H. Umezawa , H. Kawai , K. Ando , K. Takanashi , S. Maekawa , E. Saitoh , Nature 2010, 464, 262. 20220845 10.1038/nature08876

[6] A. V. Chumak , A. A. Serga , B. Hillebrands , Nat. Commun. 2014, 5, 4700. 25144479 10.1038/ncomms5700 PMC4143911

[7] A. J. Lee , J. T. Brangham , Y. Cheng , S. P. White , W. T. Ruane , B. D. Esser , D. W. McComb , P. C. Hammel , F. Yang , Nat. Commun. 2017, 8, 234. 28794430 10.1038/s41467-017-00332-x PMC5550470

[8] V. V. Kruglyak , S. O. Demokritov , D. Grundler , J. Phys. D: Appl. Phys. 2010, 43, 264001.

[9] S. Neusser , D. Grundler , Adv. Mater. 2009, 21, 2927.

[10] H. Puszkarski , M. Krawczyk , Solid State Phenom. 2003, 94, 125.

[11] P. Gruszecki , M. Krawczyk , in Wiley Encyclopedia of Electrical and Electronics Engineering (Ed: J. G. Webster), John Wiley & Sons, Ltd., New York 2016, https://onlinelibrary.wiley.com/doi/10.1002/047134608X.W8295/figures.

[12] A. V. Chumak , A. A. Serga , B. Hillebrands , J. Phys. D: Appl. Phys. 2017, 50, 244001.

[13] Z. K. Wang , V. L. Zhang , H. S. Lim , S. C. Ng , M. H. Kuok , S. Jain , A. O. Adeyeye , ACS Nano 2010, 4, 643. 20099868 10.1021/nn901171u

[14] M. Krawczyk , D. Grundler , J. Phys.: Condens. Matter 2014, 26, 123202. 24599025 10.1088/0953-8984/26/12/123202

[15] G. Venkat , N. Kumar , A. Prabhakar , IEEE Trans. Magn. 2014, 50, 7101104.

[16] S.‐K. Kim , K.‐S. Lee , D.‐S. Han , Appl. Phys. Lett. 2009, 95, 082507.

[17] H. Yu , G. Duerr , R. Huber , M. Bahr , T. Schwarze , F. Brandl , D. Grundler , Nat. Commun. 2013, 4, 2702. 24189978 10.1038/ncomms3702 PMC3831280

[18] A. A. Serga , A. V. Chumak , B. Hillebrands , J. Phys. D: Appl. Phys. 2010, 43, 264002.

[19] V. D. Bessonov , M. Mruczkiewicz , R. Gieniusz , U. Guzowska , A. Maziewski , A. I. Stognij , M. Krawczyk , Phys. Rev. B 2015, 91, 104421.

[20] M. Langer , R. A. Gallardo , T. Schneider , S. Stienen , A. Roldán‐Molina , Y. Yuan , K. Lenz , J. Lindner , P. Landeros , J. Fassbender , Phys. Rev. B 2019, 99, 024426. 10.1103/PhysRevLett.122.067204 30822086

[21] A. V. Chumak , V. S. Tiberkevich , A. D. Karenowska , A. A. Serga , J. F. Gregg , A. N. Slavin , B. Hillebrands , Nat. Commun. 2010,1, 141. 21266991 10.1038/ncomms1142 PMC3105294

[22] Z. K. Wang , V. L. Zhang , H. S. Lim , S. C. Ng , M. H. Kuok , S. Jain , A. O. Adeyeye , Appl. Phys. Lett. 2009, 94, 083112.

[23] M. Mruczkiewicz , P. Graczyk , P. Lupo , A. Adeyeye , G. Gubbiotti , M. Krawczyk , Phys. Rev. B 2017, 96, 104411.

[24] F. S. Ma , H. S. Lim , Z. K. Wang , S. N. Piramanayagam , S. C. Ng , M. H. Kuok , Appl. Phys. Lett. 2011, 98, 153107.

[25] S. Saha , R. Mandal , S. Barman , D. Kumar , B. Rana , Y. Fukuma , S. Sugimoto , Y. Otani , A. Barman , Adv. Funct. Mater. 2013, 23, 2378.

[26] A. Manzin , G. Barrera , F. Celegato , M. Cosson , P. Tiberto , Sci. Rep. 2016, 6, 22004. 26911336 10.1038/srep22004 PMC4766484

[27] J. Topp , D. Heitmann , M. P. Kostylev , D. Grundler , Phys. Rev. Lett. 2010, 104, 207205. 20867058 10.1103/PhysRevLett.104.207205

[28] K. Di , S. X. Feng , S. N. Piramanayagam , V. L. Zhang , H. S. Lim , S. C. Ng , M. H. Kuok , Sci. Rep. 2015, 5, 10153. 25950082 10.1038/srep10153 PMC4423564

[29] R. A. Gallardo , D. Cortés‐Ortuno , T. Schneider , A. Roldán‐Molina , F. Ma , R. E. Troncoso , K. Lenz , H. Fangohr , J. Lindner , P. Landeros , Phys. Rev. Lett. 2019, 122, 067204. 30822086 10.1103/PhysRevLett.122.067204

[30] L.‐L. Li , Y.‐L. Zhao , X.‐X. Zhang , Y. Sun , Chin. Phys. Lett. 2018, 35, 077401.

[31] K. Jeon , C. Ciccarelli , H. Kurebayashi , L. F. Cohen , X. Montiel , M. Eschrig , T. Wagner , S. Komori , A. Srivastava, J. W. A. Robinson , M. G. Blamire , Phys. Rev. Appl. 2019, 11, 014061.

[32] I. A. Golovchanskiy , N. N. Abramov , M. Pfirrmann , T. Piskor , J. N. Voss , D. S. Baranov , R. A. Hovhannisyan , V. S. Stolyarov , C. Dubs , A. A. Golubov , V. V. Ryazanov , A. V. Ustinov , M. Weides , Phys. Rev. Appl. 2019, 11, 044076.

[33] O. V. Dobrovolskiy , R. Sachser , T. Brächer , T. Böttcher , V. V. Kruglyak , R. V. Vovk , V. A. Shklovskij , M. Huth , B. Hillebrands , A. V. Chumak , Nat. Phys. 2019, 15, 477.

[34] A. A. Bespalov , A. S. Melnikov , A. I. Buzdin , Phys. Rev. B 2014, 89, 054516.

[35] I. A. Golovchanskiy , N. N. Abramov , V. S. Stolyarov , V. V. Bolginov , V. V. Ryazanov , A. A. Golubov , A. V. Ustinov , Adv. Funct. Mater. 2018, 28, 1802375.

[36] I. A. Golovchanskiy , N. N. Abramov , V. S. Stolyarov , V. V. Ryazanov , A. A. Golubov , A. V. Ustinov , J. Appl. Phys. 2018, 124, 233903.

[37] T. Yukawa , J.‐I. Yamada , K. Abe , J.‐I. Ikenoue , Jpn. J. Appl. Phys. 1977, 16, 2187.

[38] M. Mruczkiewicz , M. Krawczyk , J. Appl. Phys. 2014, 115, 113909.

[39] B. Lebed , S. Yzkovlev , Pisma v ZhTF (in Russian) 1989, 15, 27.

[40] V. B. Anfinogenov , Y. V. Gulyaev , P. E. Zilberman , I. M. Kotelyanskiy , N. I. Polzikova , A. A. Suhanov , Pisma v ZhTF (in Russian) 1989, 15, 24.

[41] D. Stancil , Theory of Magnetostatic Waves, Springer‐Verlag New York, Inc., New York 1993.

[42] I. Neudecker , G. Woltersdorf , B. Heinrich , T. Okuno , G. Gubbiotti , C.H. Back , J. Magn. Magn. Mater. 2006, 307, 148.

[43] S. S. Kalarickal , P. Krivosik , M. Wu , C. E. Patton , M. L. Schneider , P. Kabos , T. J. Silva , J. P. Nibarger , J. Appl. Phys. 2006, 99, 093909.

[44] Y.‐C. Chen , D.‐S. Hung , Y.‐D. Yao , S.‐F. Lee , H.‐P. Ji , C. Yu , J. Appl. Phys. 2007, 101, 09C104.

[45] I. A. Golovchanskiy , V. V. Bolginov , N. N. Abramov , V. S. Stolyarov , A. Ben Hamida , V. I. Chichkov , D. Roditchev , V. V. Ryazanov , J. Appl. Phys. 2016, 120, 163902.

[46] V. V. Schmidt , The Physics of Superconductors. Introduction to Fundamentals and Applications, Springer‐Verlag, Berlin, Heidelberg 1997.

[47] C. P. Bean , Rev. Mod. Phys. 1964, 36, 31.

[48] W. T. Norris , J. Phys. D: Appl. Phys. 1969, 3, 489.

[49] M. Willemin , C. Rossel , J. Hofer , H. Keller , A. Erb , E. Walker , Phys. Rev. B 1998, 58, R5940.

[50] E. H. Brandt , G. P. Mikitik , Phys. Rev. Lett. 2002, 89, 027002. 12097012 10.1103/PhysRevLett.89.027002

[51] G. P. Mikitik , E. H. Brandt , Phys. Rev. B 2004, 69, 134521.

[52] N. Avraham , B. Khaykovich , Y. Myasoedov , M. Rappaport , H. Shtrikman , D. E. Feldman , T. Tamegai , P. H. Kes , M. Li , M. Konczykowski , K. van der Beek , E. Zeldov , Nature 2001, 411, 451. 11373671 10.1038/35078021

[53] I. A. Golovchanskiy , A. V. Pan , J. George , F. S. Wells , S. A. Fedoseev , A. Rozenfeld , Supercond. Sci. Technol. 2016, 29, 075002.

[54] I. A. Golovchanskiy , A. V. Pan , T. H. Johansen , J. George , I. A. Rudnev , A. Rozenfeld , S. A. Fedoseev , Phys. Rev. B 2018, 97, 014524.

[55] P. Deorani , J. H. Kwon , H. Yang , Curr. Appl. Phys. 2014, 14, S129.

[56] M. Jamali , J. H. Kwon , S.‐M. Seo , K.‐J. Lee , H. Yang , Sci. Rep. 2013, 3, 3160. 24196318 10.1038/srep03160 PMC3819604

[57] M. Mruczkiewicz , M. Krawczyk , G. Gubbiotti , S. Tacchi , Y. A. Filimonov , D. V. Kalyabin , I. V. Lisenkov , S. A. Nikitov , New J. Phys. 2013, 15, 113023.

[58] M. Mruczkiewicz , E. S. Pavlov , S. L. Vysotsky , M. Krawczyk , Y. A. Filimonov , S. A. Nikitov , Phys. Rev. B 2014, 90, 174416.

[59] M. Donahue , D. Porter , OOMMF User's Guide, Version 1.0, Interagency Report NISTIR 6376, National Institute of Standards and Technology, Gaithersburg, MD 1999.

[60] J. E. Miltat , M. J. Donahue , in Handbook of Magnetism and Advanced Magnetic Materials (Ed: H. Kronmueller, S. Parkin), Vol. 2, John Wiley & Sons, Ltd., New York 2007, https://onlinelibrary.wiley.com/doi/abs/10.1002/9780470022184.hmm202.

[61] G. Venkat , D. Kumar, M. Franchin , O. Dmytriiev , M. Mruczkiewicz , H. Fangohr , A.Barman, M. Krawczyk , A. Prabhakar , IEEE Trans. Magn. 2013, 49, 524.

[62] S.‐K. Kim , J. Phys. D: Appl. Phys. 2010, 43, 264004.

[63] M. Dvornik , Y. Au , V. V. Kruglyak , Top. Appl. Phys. 2013, 125, 101.

