# Supplementary material for: Ferromagnet/Superconductor Hybrid Magnonic Metamaterials
Source: Adv Sci (Weinh). 2019 Jul 6;6(16):1900435. doi: 10.1002/advs.201900435 (PMC6702653; doi:10.1002/advs.201900435)
Supplement: Supplementary file 1 — Supplementary [file ADVS-6-1900435-s001.pdf]

Copyright WILEY-VCH Verlag GmbH & Co. KGaA, 69469 Weinheim, Germany, 2019.

ADVANCED SCIENCE
Open Access

Supporting Information

for *Adv. Sci.,* DOI: 10.1002/advs.201900435

Ferromagnet/Superconductor Hybrid Magnonic Metamaterials

Igor A. Golovchanskiy, Nikolay N. Abramov, Vasily S. Stolyarov, Pavel S. Dzhumaev, Olga V. Emelyanova, Alexander A. Golubov, Valery V. Ryazanov, and Alexey V. Ustinov*

Supplementary materials for

Ferromagnet/superconductor hybrid magnonic metamaterials

I. A. Golovchanskiy,[1,2] N. N. Abramov,[2] V. S. Stolyarov,[1,3,4] P. S. Dzhumaev,[5] O. V. Emelyanova,[5] A. A. Golubov,[1,6] V. V. Ryazanov,[2,3,4] and A. V. Ustinov[2,7]

[1)] *Moscow Institute of Physics and Technology, National Research University, 9 Institutskiy per., Dolgoprudny, Moscow Region, 141700, Russia.*

[2)] *National University of Science and Technology MISIS, 4 Leninsky prosp., Moscow, 119049, Russia.*

[3)] *Institute of Solid State Physics (ISSP RAS), Chernogolovka, 142432, Moscow region, Russia.*

[4)] *Solid State Physics Department, Kazan Federal University, 420008 Kazan, Russia.*

[5)] *National Research Nuclear University MEPhI (Moscow Engineering Physics Institute), 31 Kashirskoye Shosse, 115409, Moscow, Russia.*

[6)] *Faculty of Science and Technology and MESA+ Institute for Nanotechnology, University of Twente, 7500 AE Enschede, The Netherlands.*

[7)] *Physikalisches Institut, Karlsruhe Institute of Technology, 76131 Karlsruhe, Germany.*

SEM of the investigated sample

Figure 1 shows scanning electron microscopy (SEM) images of the investigated sample taken with the tilted sample table. Figure 1a provides a general view on the central transmission line of Nb coplanar waveguide with the hybrid MC on top of it. Brighter contrast of Nb stripes as compared to Nb coplanar is generated by the charging effect of electrically isolated stripes. Figure 1b highlights the triangular cross-section shape of superconducting Nb stripes. The triangular cross-section is textured by a shadow effect that is formed by the off-axis deposition of sputtered Nb on the substrate, which is primed for the lift-off process, (i.e., covered with a patterned photo-resist).

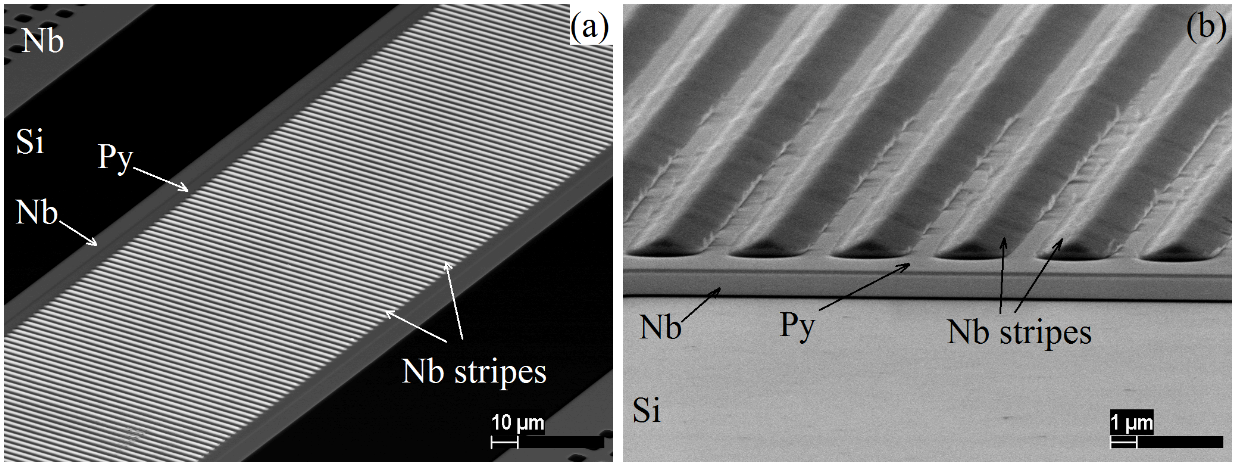

FIG. 1. a) SEM image of the fabricated structure that is placed on top of the central line of Nb co-planar waveguide. The image is taken with the back-scattered electrons and with the tilt angle of the substrate table 60°. b) Magnified SEM image of the fabricated structure taken with the secondary electrons and with the tilt angle 84°. Triangular shape of Nb stripes is well distinguishable.

FMR spectroscopy of hybrid metamaterials

Figure 2 compares FMR spectra of the pristine sample and MC samples in both the backward-volume and the surface spin-wave geometries measured at $T = 4$ K$< T_c$ and $T = 10$ K$> T_c$. FMR spectrum of the pristine sample at $T = 4$ K (Fig. 2a) shows the typical Kittel resonance frequency-field dependence for thin ferromagnetic films in in-plane

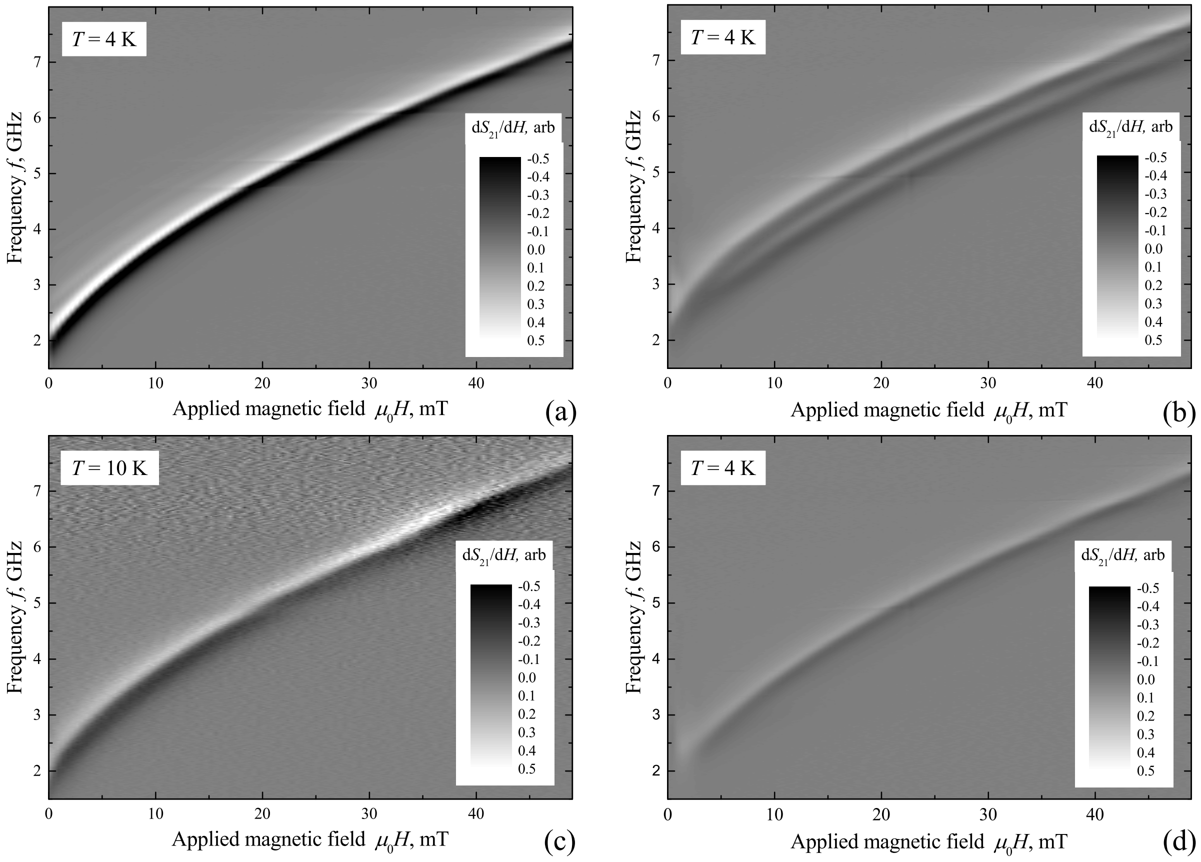

FIG. 2. Transmission spectra $dS_{21}(f,H)/dH$ of the pristine permalloy film measured at a) The spectrum of the pristine permalloy film at $T = 4$ K. b) The spectrum of the MC at BV geometry at $T = 4$ K. c) The spectrum of the MC at BV geometry at $T = 10$ K. The sample in (b) and (c) is formed by placing Nb stripes on the same Py film as in (a). d) The spectrum of the MC at MSSW geometry at $T = 4$ K.

magnetic fields. FMR spectrum of the MC sample in the BV geometry at $T = 4$ K$< T_c$ (Fig. 2b) displays a split of the FMR signal into two spectral lines. FMR spectrum of the same MC sample at $T = 10$ K$> T_c$ (Fig. 2c) shows a single FMR line and in general reproduces one of the pristine sample in Fig. 2a. This temperature dependence of the FMR spectrum indicates that the splitting of FMR signal at temperatures below T_c of Nb stripes is associated with superconductivity of Nb stripes. FMR signal at 10 K in (Fig. 2c) is more noisy than one at 4 K in Figs. 2a,b. We attribute this effect to a finite conductance of Nb CPW at normal state.

Figure 2d shows FMR spectrum of a different MC sample in the MSSW geometry at

$T = 4$ K$< T_c$. In Fig. 2d the superconducting periodic structure is represented by a similar array of stripes of dimensions $X \times Y \times Z = 50 \times 3 \times 0.7\ \mu\text{m}^3$ located with the same period $a = 4\ \mu$m along y-axis (see Fig. 1 in the main text.) The array of stripes is placed on top of $50 \times 130\ \mu\text{m}^2$ Py rectangle film of thickness $d = 50$nm. The Py/Nb sample is placed on top of the central stripe of the same superconducting Nb coplanar waveguide. FMR spectrum of the MC in MSSW geometry at $T < T_c$ in Fig. 2d shows no signature of the interplay of magnetization dynamics with superconducting stripes. When magnetic field is applied along Nb stripes the stray field in Py produced by diamagnetism of Nb stripes is negligible due to their small demagnetising factor. Therefore, unlike in case of the BV geometry, in MSSW geometry periodic modulation of the DC magnetic field in Py is absent. One may consider the AC effect when superconducting stripes screen AC stray fields of precessing magnetic moments. However, the Kittel-FMR in thin films depicts uniform coherent precession of all magnetic moments in the entire film, which implies absence of AC stray fields outside of ferromagnetic film. Absence of the effect in MSSW geometry proves that the interplay of magnetization dynamics with superconducting stripes is purely magnetostatic.
